# Supplementary material for: Evolution of Youth’s Mental Health and Quality of Life during the COVID-19 Pandemic in South Tyrol, Italy: Comparison of Two Representative Surveys
Source: Children (Basel). 2023 May 17;10(5):895. doi: 10.3390/children10050895 (PMC10217242; doi:10.3390/children10050895)
Supplement: Supplementary file 1 [file children-10-00895-s001.zip › children-2382555-supplementary.pdf]

Supplementary Material

Table S1. Results of the stepwise linear regression models

Item	Report	COP-S Survey	Predictor	Regression Coefficient Beta	Confidence Interval	p-value
HRQoL [1]	Proxy-reported	2021	older age	-0.334	[-0.431;-0.237]	< 0.001
			single parenthood	-1.286	[-2.503;-0.069]	0.038
			elevated parental burden due to the pandemic	-1.597	[-2.281;-0.913]	< 0.001
			low family climate	-7.680	[-8.398;-6.962]	< 0.001
			elevated burden of school	-4.439	[-5.144;-3.734]	< 0.001
			extended use of digital media	-2.919	[-3.684;-2.154]	< 0.001
			less contact with friends	-3.234	[-4.028;-2.440]	< 0.001
		2022	older age	-0.370	[-0.454;-0.286]	< 0.001
			single parenthood	-1.142	[-2.201;-0.083]	0.035
			elevated parental burden due to the pandemic	-1.068	[-1.682;-0.454]	= 0.001
			low family climate	-6.899	[-7.581;-6.218]	< 0.001
			elevated burden of school	-4.281	[-4.895;-3.668]	< 0.001
			extended use of digital media	-2.672	[-3.300;-2.045]	< 0.001
			less contact with friends	-3.060	[-3.672;-2.448]	< 0.001
	Self-reported	2021	Female gender x older age	-0.175	[-0.238;-0.113]	< 0.001
			Migration background	-1.761	[-3.244;-0.278]	0.020
			elevated parental burden due to the pandemic	-1.680	[-2.641;-0.720]	0.001
			low family climate	-7.369	[-8.443;-6.295]	< 0.001
			elevated burden of school	-3.730	[-4.743;-2.717]	< 0.001
			extended use of digital media	-1.716	[-2.744;-0.689]	0.001
			Less contact with friends	-4.376	[-5.359;-3.393]	< 0.001
			gender x age	-0.120	[-0.179;-0.062]	< 0.001
			good family climate	-6.874	[-8.006;-5.741]	< 0.001
			no elevated burden of school	-4.472	[-5.368;-3.577]	< 0.001
			good contact with friends	-3.833	[-4.748;-2.919]	< 0.001
Symptoms of anxiety	self-reported	2021	older age x female gender	0.148	[0.120;0.177]	< 0.001

			elevated parental burden due to the pandemic	0.728	[0.299;1.156]	0.001
			lower family climate	2.240	[1.761;2.720]	< 0.001
			elevated burden of school	1.056	[0.605;1.507]	< 0.001
			less contact with friends	0.988	[0.557;1.420]	< 0.001
		2022	older age x female gender	0.117	[0.091;0.143]	< 0.001
			elevated parental burden due to the pandemic	0.630	[0.222;1.039]	0.003
			lower family climate	2.611	[2.100;3.121]	< 0.001
			elevated burden of school	1.210	[0.805;1.616]	< 0.001
			less contact with friends	0.962	[0.550;1.374]	< 0.001
Depressive symptoms	self-reported	2021	older age	0.092	[0.065;0.119]	< 0.001
			older age x female gender	0.027	[0.019;0.036]	< 0.001
			elevated parental burden due to the pandemic	0.150	[0.021;0.279]	0.023
			lower family climate	0.992	[0.847;1.137]	< 0.001
			elevated burden of school	0.267	[0.132;0.402]	< 0.001
			less contact with friends	0.273	[0.143;0.403]	< 0.001
		2022	older age	0.090	[0.064;0.115]	< 0.001
			older age x female gender	0.018	[0.010;0.026]	< 0.001
			lower family climate	0.928	[0.773;1.082]	< 0.001
			elevated burden of school	0.358	[0.235;0.480]	< 0.001
			less contact with friends	0.385	[0.260;0.509]	< 0.001
Psychosomatic symptoms	proxy-reported	2021	olderer age	-0.178	[-0.265;-0.091]	< 0.001
			older age x female gender	-0.068	[-0.096;-0.040]	< 0.001
			single parenthood	-1.114	[-2.098;-0.130]	0.027
			elevated parental burden due to the pandemic	-1.084	[-1.506;-0.662]	< 0.001
			low family climate	-3.076	[-3.547;-2.605]	< 0.001
			elevated burden of school	-1.030	[-1.465;-0.595]	< 0.001
		2022	older age x female gender	-0.061	[-0.086;-0.035]	< 0.001
			Single parenthood	-1.372	[-2.236;-0.508]	0.002
			Migration background	-0.780	[-1.430;-0.131]	0.019
			elevated parental burden due to the pandemic	-0.613	[-1.010;-0.215]	0.003
			low family climate	-2.508	[-3.009;-2.006]	< 0.001
			Less contact with friends	-0.893	[--1.292;--0.493]	< 0.001
			elevated burden of school	-1.231	[-1.623;-0.840]	< 0.001
	self-reported	2021	older age	-0.263	[-0.371;-0.154]	< 0.001

Psychosomatic symptoms		older age x female gender	-0.141	[-0.176;-0.106]	< 0.001
		single parenthood	-1.352	[-2,277;-0.428]	< 0.001
		elevated parental burden due to the pandemic	0.788	[-1.315;-0.261]	< 0.001
		low family climate	-3.461	[-4.050;-2.873]	< 0.001
		elevated burden of school	-0.919	[-1.462;-0.377]	< 0.001
	2022	older age x female gender	-0.355	[-0.496;-0.215]	< 0.001
		female gender	3.520	[1.447;5.594]	< 0.001
		low family climate	-3.704	[-4.309;-3.099]	< 0.001
		elevated burden of school	-1.506	[-1.985;-1.027]	< 0.001

[1] HRQoL, health-related quality of life.
